# Comparison of fidaxomicin, metronidazole and vancomycin for initial episode and recurrence of *Clostridioides difficile* infection - An observational cohort study

**DOI:** 10.1016/j.heliyon.2024.e30742

**Published:** 2024-05-07

**Authors:** Marcos Hernando-Gozalo, Carlos Rescalvo-Casas, Laura Seijas-Pereda, Juan Cuadros-González, Ramón Pérez-Tanoira

**Affiliations:** a Universidad de Alcalá, Departamento de Química Orgánica y Química Inorgánica, Instituto de Investigación en Química “Andrés M. del Río” (IQAR), 28805, Madrid, Spain; b Departamento de Microbiología Clínica, Hospital Universitario Príncipe de Asturias, 28805, Madrid, Spain; c Universidad de Alcalá, Facultad de Medicina, Departamento de Biomedicina y Biotecnología, 28805, Madrid, Spain

**Keywords:** *Clostridioides difficile*, Vancomycin, Metronidazole, Fidaxomicin, Hypervirulent *Clostridioides difficile*, Recurrent *Clostridioides difficile* infection

## Abstract

**Objectives:**

The main aim of this study was to compare the clinical outcomes of patients attended in our area with *Clostridioides difficile* infection (CDI) (sustained cure, recurrence or death) in relation to treatment to normal or hypervirulent *C. difficile* as a risk factor and to describe the resistance profile to metronidazole and vancomycin antibiotics in our hospital over a one-year period.

**Methods:**

A retrospective, cross-sectional and observational study was conducted between June 2022 and June 2023 to compare the clinical cure and/or recurrence of CDI in adult patients treated in a Spanish secondary Hospital depending on the prescribed antibiotic treatment. In addition, we performed an antimicrobial susceptibility study to vancomycin and metronidazole in all *C. difficile* isolated in bacterial culture.

**Results:**

Out of 194 selected patients the treatments were as follow: 43.81 % vancomycin, 21.65 % metronidazole, 8.25 % a combination of both, 6.70 % fidaxomicin and 19.59 % were untreated. Vancomycin and fidaxomicin patients had higher odds ratio of prolonged hospitalization (*p* = 0.041 and *p* = 0.040, respectively). Fidaxomicin had increased odds of suffering another episode of *C. difficile* (*p* = 0.009) and it was inferior to metronidazole for recurrent CDI (rCDI) (*p* = 0.035).

Resistance profile for *C. difficile* was 4.07 % for vancomycin and 3.49 % for metronidazole. Hypervirulent *C. difficile* was identified in 17 (8.76 %) patients with 29.41 % of mortality (5/17; p > 0.05).

**Conclusion:**

Fidaxomicin treated patients had statistically increased odds of rCDI. Compared to other treatments, fidaxomicin was inferior to metronidazole for rCDI in our cohort;

Hypervirulent *C. difficile* was not associated with death.

Vancomycin resistance of *C. difficile* statistically decreased, whereas metronidazole resistance did not vary during the studied period.

## Highlights

• In our cohort, metronidazole was superior to fidaxomicin for sustained clinical response and for avoiding recurrences after the first episode of CDI.
• The percentage of resistance to metronidazole and vancomycin was below 5 % in the strains studied.
• Hypervirulent *C. difficile* was not associated to death or of suffering recurrent *C. difficile* infection.

## 1 Introduction

*Clostridioides difficile* stands as the leading cause of nosocomial (hospital-acquired) diarrhea in adult patients in developed countries, and its role as a causative agent in community-acquired diarrhea is on the rise [[1], [2], [3]].

Currently, diagnosis of *Clostridioides difficile* infection (CDI) is carried out by detection of toxins A and/or B in stool samples by enzyme immunoassay [3]. As an initial method, detection of glutamate dehydrogenase (GDH) by immunoassay is used because of its high diagnostic sensitivity (≈90 %). Positive results are subsequently confirmed using enzyme immunoassays and molecular techniques, which exhibit higher sensitivity and specificity [3].

An epidemiological report from the European Centre for Disease Prevention and Control noted 7.711 cases of CDI in the European Union/European Economic Area in 2016 [4]. Of these cases, 74.7 % were nosocomial, 7.9 % were relapses and 16.7 % had a complicated clinical course [4].

Antimicrobial resistance is a serious public health problem recognized by the World Health Organisation (WHO), with important health and socio-economic consequences. Nowadays, drugs used against CDI include metronidazole, vancomycin and fidaxomicin.

Metronidazole is a nitroimidazole against anaerobic bacteria and protozoa with a rapid bactericidal effect depending on the concentration by causing oxidative DNA damage [5]. Vancomycin is a glycopeptide with slow bactericidal activity that blocks the synthesis of cell wall peptidoglycan [5]. Fidaxomicin is a macrolide that inhibits bacterial RNA polymerase, thereby halting RNA synthesis [5]. This mechanism alters gene expression of *C. difficile* toxin and sporulation genes [5].

The European Society of Clinical Microbiology and Infectious Diseases (ESCMID), the Infectious Diseases Society of America (IDSA) and the Society for Healthcare Epidemiology of America (SHEA) recommend fidaxomicin as the first treatment of choice for the initial episode or CDI recurrences when available and feasible [6,7]. Because of its implementation depends upon available resources, vancomycin remains an acceptable alternative. However, several studies support that there is no benefit of using vancomycin compared to metronidazole for treating initial episode of CDI [[8], [9], [10]]. Alternatively, other studies suggest that fidaxomicin is more effective than metronidazole and vancomycin, particularly in eradicating an initial episode [11,12] or preventing CDI recurrence [13].

Antimicrobial resistance to metronidazole, vancomycin and fidaxomicin has been reported, although it remains uncommon [14,15]. According to the meta-analysis by Sholeh et al. (2020) [16], resistance to metronidazole and vancomycin is 1 % worldwide. On the other hand, Boyanova et al. (2023) [17], performed a study including 20 different countries where they found 4.7 % and 2.6 % resistance strains for metronidazole and vancomycin, respectively.

The most relevant risk factors with CDI include previous antibiotic use, age over 65 years and hospitalization [1,8]. Other potential contributing factors are immunosuppression or gastrointestinal and non-gastrointestinal co-infections [8]. Therefore, a better understanding of potential patient risk factors and antimicrobial resistance would allow for optimal early treatment.The main aim of this study was to compare the clinical outcomes of patients attended in our area with CDI (sustained cure, recurrence, or death) in relation to CDI treatment. Additionally, we investigated the potential impact of *Clostridioides difficile* ribotype 027 as a risk factor for mortality or recurrence in CDI. Furthermore, our last objective was to analyse the antimicrobial susceptibility profile and the variations in the minimum inhibitory concentrations of vancomycin and metronidazole over a one-year period of *C. difficile* strains isolated from these patients.

## 2 Material and methods

### 2.1 Patients

This observational cohort study was carried out between June 2022 and June 2023 in the Microbiology Department of the Hospital Universitario Príncipe de Asturias, Alcalá de Henares, Spain, counting on 417 beds and catering for a 243.053 inhabitant area. We have reviewed the medical records of the patients with CDI confirmed by the Clinical Microbiology laboratory using a chemiluminescent immunoassay (CLIA) intended for the determination of glutamate dehydrogenase (GDH) and toxins A/B on the LIAISON Analyzer (DiaSorin S.p.A. Italy) in human faeces. On the other hand, the molecular assay multiplex Seegene Allplex TM GI-Bacteria (I and II) (Seegene Diagnostics, Seoul, South Korea; Seegene) ands Xpert C. difficile BT Assay (Cepheid AB, Solna, Sweden) were used to differentiate strains with ribotype 027. A positive case was defined as any patient showing diarrhoeal stools (≥3 non-formed stools/24 h), accompanied by a positive laboratory result for the presence of toxin A and/or B in stool or positive PCR with a cycle threshold less than 40 [18,19].

Patients consistent with the positive case definition outlined above, who were at least 18 years of age, were included. Day-cases such as outpatient surgery patients with 24-h hospital admission and patients on in-hospital dialysis were excluded [19]. In addition, patients under 18 years old were exclusively studied for *C. difficile* antimicrobial susceptibility and were not included in the epidemiological study.

Hospital-acquired or nosocomial infections were defined as the acquisition of a disease/pathogen 48 h after being hospitalised without any evidence of previous infection [19].

Recurrence of CDI was defined by ESCMID and IDSA criteria as the reappearance of symptoms within eight weeks after a previous episode, provided the symptoms form the previous episode resolved after completion of initial treatment [6,7,20].

### 2.2 Study population

We reviewed the history of CDI episodes up to ten years before. For epidemiological analysis, a minimum follow-up of three months had to be performed in order to find possible relapses and/or re-infections [8].

Studies were conducted based on patient demographics and the effect of treatments on patients. For these reasons, we divided the patients as follows: no treatment, metronidazole, vancomycin, fidaxomicin and combination (vancomycin + metronidazole).

### 2.3 Study variables

Clinical data included demographic variables (age, sex), epidemiological variables (nosocomial infection), clinical variables (hospitalization, admission to the Intensive Care Unit (ICU), clinical course, previous *C. difficile* infection, gastrointestinal comorbidity, immunosuppression, Proton-pump inhibitors (PPIs) use, prescribed treatment, change of treatment and death), and microbiological variables (microbiological tests performed, gastrointestinal or non-gastrointestinal coinfections and antimicrobial susceptibility).

### 2.4 Antibiotic resistance study

The antimicrobial susceptibility study included the 172 viable *C. difficile* strains isolated from CDI diagnosed patients between June 2022 and 2023.First, samples in which *C. difficile* was detected were seeded on chromID® *C. difficile* (CDIF) selective agar medium (Biomérieux, Îlle de France, France). After incubation under anaerobic conditions at 37 °C for 24 h, C. difficile colonies were identified by matrix-assisted laser desorption/ionization time-of-flight (MALDI-TOF) mass spectrometry using the VITEK® MS system (Biomériux, Lyon, France).

The minimum inhibitory concentration (MIC) was determined for metronidazole and vancomycin on CDIF agar using a gradient strip test (Etest) (Biomérieux, Marcy-l’Étoile, France) [21]. The strains were classified as susceptible or resistant according to the European Committee on Antimicrobial Susceptibility Testing (EUCAST) guidelines. Thus, strains were considered sensitive or resistant for metronidazole and vancomycin, if they had respectively MIC ≤2 mg/L or MIC >2 mg/L [22].

### 2.5 Statistical analysis

Continuous variables are presented as median and interquartile range (IQR) and categorical variables as percentages. Comparisons between continuous variables were made using Student's t-test or the Mann-Whitney test, depending on the normality of the distribution. Differences between categorical variables were made using the two-tailed Fisher's exact test. For comparisons, a p-value ≤0.05 was considered significant. Statistical analysis was performed with SPSS v20.0 (IBM Corp., Armonk, NY, USA).

Univariate or multivariate logistic regression models analysed the effectiveness of the different treatments logistic regression model with results expressed as odds ratio (OR) and 95 % confidence intervals (95 % CI).

The logistic regression model was adjusted for the most significant variables, with results expressed as odds ratios (OR) and 95 % confidence intervals (CI).

## 3 Results

### 3.1 Demographic description of patients

The profile of patients according to the antibiotic dispensed for the treatment of the first episode CDI is shown in Table 1. During the 12-month study, 142 patients were hospitalised with CDI; 104 were females (53.61 %); 35 patients died (18.04 %). The median age was 77.50 years (IQR: 64.75–87.00). Sixty-one patients had a recurrent CDI (31.44 %). Ten patients (5.15 %) were admitted to an intensive care unit.

**Table 1:** Patient profile according to the medication prescribed for first episode *Clostridioides difficile* infection (CDI).

	**No treatment (n = 38)**	**Metronidazole (n = 42)**	**Vancomycin (n = 85)**	**Fidaxomicin (n = 13)**	**Combined (n = 16)**^a^	**All patients (n = 194)**
Age **(median)**	73.50 (58.75–87.00)	77.00 (55.00–87.00)	76.00 (64.50–87.00)	83.00 (75.50–87.00)	88.00 (76.25–92.00)	77.50 (64.75–87.00)
**Sex (male)**	23 (60.53 %)	19 (45.24 %)	34 (40.00 %)	6 (46.15 %)	8 (50.00 %)	90 (46.39 %)
**Hospitalization**	12 (31.58 %)	24 (57.14 %)	78 (91.76 %)	13 (100.00 %)	15 (93.75 %)	142 (73.20 %)
**Days** hospitalised	0 (0–5)	5.50 (0–20)	11 (6.5–18.5)	22 (9–30.5)	27 (13–37.75)	9 (0.25–20)
**Nosocomial**	8 (21.05 %)	12 (28.57 %)	40 (47.06 %)	8 (61.54 %)	10 (62.50 %)	78 (40.21 %)
**I**ntensive Care Unit **(ICU)**	1 (2.63 %)	1 (2.38 %)	7 (8.24 %)	0 (0.00 %)	1 (6.25 %)	10 (5.15 %)
**Death**	7 (18.42 %)	10 (23.81 %)	12 (14.12 %)	2 (15.38 %)	4 (25.00 %)	35 (18.04 %)
**Proton-pump inhibitors (PPIS)**	20 (52.63 %)	21 (50.00 %)	64 (75.29 %)	11 (84.62 %)	13 (81.25 %)	129 (66.49 %)
**Recurrence**	5 (13.16 %)	11 (26.19 %)	31 (36.47 %)	8 (61.54 %)	6 (37.50 %)	61 (31.44 %)
**Treatment change**	1 (2.63 %)	9 (21.43 %)	13 (15.29 %)	3 (23.08 %)	2 (12.50 %)	28 (14.43 %)
**GI C**oinfection**	6 (15.79 %)	5 (11.90 %)	7 (8.24 %)	0 (0.00 %)	0 (0.00 %)	18 (9.28 %)
No GI Coinfection **	5 (13.16 %)	10 (23.81 %)	34 (40.00 %)	3 (23.08 %)	9 (56.25 %)	61 (31.44 %)
GI Comorbidity**	22 (57.89 %)	22 (52.38 %)	43 (50.59 %)	9 (69.23 %)	5 (31.25 %)	101 (52.06 %)
**Immunosuppression**	10 (26.32 %)	12 (28.57 %)	37 (43.53 %)	4 (30.77 %)	8 (50.00 %)	71 (36.60 %)

a **Combined treatment**: Vancomycin + Metronidazole** GI: Gastrointestinal.

The antibiotic regimens and the number of patients were as follows: 85 (43.81 %) vancomycin, 42 (21.65 %) metronidazole, 16 (8.25 %) a combination of both, 13 (6.70 %) fidaxomicin and, 38 (19.59 %) were untreated.

Table 2 shows the results obtained by performing the chi-square and multivariate tests for the variables: Age, Sex, Hospitalization, Days hospitalised, Nosocomial, ICU, Death, Recurrence, GI Coinfection, No GI Coinfection, GI Comorbidity and Inmunosupression.

**Table 2:** Chi-square test and multivariate analysis for possible risk factors according to the medication prescribed for first episode *Clostridioides difficile* infection (CDI).

	**Treatment/No treatment (n = 194)**			**Metronidazole (n = 42)**			**Vancomycin (n = 85)**			**Fidaxomicin (n = 13)**		
	**X**^**2**^	**OR Multivariate**	**p-value**	**X**^**2**^	**OR Multivariate**	**p-value**	**X**^**2**^	**OR Multivariate**	**p-value**	**X**^**2**^	**OR Multivariate**	**p-value**
**Age**	0.116	–		0.599	–	–	0.861	–	–	0.226	–	–
**Sex**	0.051	–		0.866	–	–	0.115	–	–	0.986	–	–
**Hospitalization**	**<0.001**	**4.543 (1.256–15.439)**	**0.021**	**0.008**	**0.384 (0.187–0.790)**	**0.009**	**<0.001**	2.716 (0.816–9.040)	0.103	**0.024**	2.727 (0.818–9.087)	0.102
**Days hospitalised**	**<0.001**	**1.106 (1.012–1.209)**	**0.026**	0.096	–	–	**0.007**	**1.088 (1.003–1.179)**	**0.041**	**0.008**	**1.094 (1.004–1.192)**	**0.040**
**Nosocomial**	**0.007**	0.374 (0.109–1.286)	0.118	0.082	–	–	0.086			0.104	–	–
**ICU**^a^	0.449	–	–	0.355	–	–	0.089	–	–	0.383	–	–
**Death**	0.946	–	–	0.844	–	–	0.209	–	–	0.796	–	–
**Recurrence**	0.007	–	–	0.407	–	–	0.183	–	–	**0.016**	**6.866 (1.605–29.373)**	**0.009**
**GI Coinfección**^b^	0.123	–	–	0.507	–	–	0.658	–	–	0.233	–	–
**No GI Coinfection**^b^	0.091	–	–	0.229	–	–	**0.023**	1.951 (0.576–6.600)	0.283	0.501	–	–
**GI Comorbidity**^b^	0.422	–	–	0.963	–	–	0.717	–	–	0.200	–	–
**Inmunosupression**	0.142	–	–	0.222	–	–	0.077			0.652	–	–

a ICU: Intensive Care Unit.

b GI: Gastrointestinal.

Regarding patients who received treatment, they had a higher odds ratio of hospitalization and for longer periods compared to those who did not receive treatment (*p* = 0.021 and *p* = 0.026, respectively) (Table 2).

Patients treated with metronidazole had lower odds ratio of being hospitalised (*p* = 0.009) and not showed any increased odds ratio for any of the variables (Table 2). According to patients who received vancomycin or fidaxomicin, they had a higher odds ratio of prolonged hospitalization with statistically significant differences (*p* = 0.041 and *p* = 0.040, respectively). Meanwhile, patients treated with fidaxomicin had statistically increased odds of suffering another episode of *C. difficile* (*p* = 0.009) (Table 2).

### 3.2 Treatment comparison

#### 3.2.1 First episode of *Clostridiodes difficile* infection (CDI)

Non-treated patients were not included for the treatment comparison. Therefore, the analysed group included 156 patients, 28 of them died (17.95 %). Meanwhile, of the remaining 128, 56 patients developed recurrent recurrent *Clostridioides difficile* infection rCDI (43.75 %).

One the one hand, neither the drugs used, nor their combination showed an increased odds ratio of sustained cure of the first episode of CDI (Table 3) (Supplementary Fig. 1). On the other hand, significant differences were found only in patients with rCDI who received metronidazole versus those treated with fidaxomicin [OR = 5.091 (95%IC: 1.120–23.142); *p* = 0.035] (Table 4) (Supplementary Fig. 1).

**Table 3:** Comparison of effectiveness of different treatments for *Clostridioides difficile* cure. Recurrences were compared using univariate regression analysis.CDI: *Clostridioides difficile* infection; OR: odds ratio; CI: confidential interval; MTZ: metronidazole; VAN: vancomycin FDX: fidaxomicin.

	**MTZ/VAN**		**MTZ/FDX**		**MTZ/MTZ + VAN**		**VAN/FDX**		**VAN/MTZ + VAN**		**FDX/MTZ + VAN**	
	**OR (95%CI)**	**p-Value**	**OR (95%CI)**	**p-Value**	**OR (95%CI)**	**p-Value**	**OR (95%CI)**	**p-Value**	**OR (95%CI)**	**p-Value**	**OR (95%CI)**	**p-Value**
**1stEpisode**	1.024(0.489–2.144)	0.950	3.333 (0.802–13.859)	0.098	1.667 (0.513–5.419)	0.396	3.256 (0.837–12.666)	0.089	1.750 (0.582–5.262)	0.319	2.000 (0.388–10.309)	0.407
**2ndEpisode**	1.128 (0.206–6.168)	0.889	0.889 (0.144–5.479)	0.889	1.333 (0.057–31.121)	0.858	0.788 (0.213–2.915)	0.721	0.846 (0.047–15.161)	0.910	0.667 (0.035–12.840)	0.788

**Table 4:** Comparison of effectiveness of different treatments for recurrent *Clostridioides difficile* infection (rCDI). Recurrences were compared using univariate regression analysis. CDI: *Clostridioides difficile* infection; OR: odds ratio; CI: confidential interval; FDX: fidaxomicin; MTZ: metronidazole; VAN: vancomycin.

	**MTZ/VAN**		**MTZ/FDX**		**MTZ/MTZ + VAN**		**VAN/FDX**		**VAN/MTZ + VAN**		**FDX/MTZ + VAN**	
	**OR (95%CI)**	**p-Value**	**OR (95%CI)**	**p-Value**	**OR (95%CI)**	**p-Value**	**OR (95%CI)**	**p-Value**	**OR (95%CI)**	**p-Value**	**OR (95%CI)**	**p-Value**
**1**^**st**^**Episode**	1.409 (0.594–3.345)	0.437	5.091 (1.120–23.142)	**0.035**	1.909 (0.497–7.276)	0.347	3.613 (0.886–14.735)	0.073	1.500 (0.439–5.124)	0.518	2.667 (0.466–15.252)	0.270
**2**^**nd**^**Episode**	1.533 (0.137–17.334)	0.727	1.778 (0.148–21.395)	0.650	4.000 (0.117–136.957)	0.442	1.156 (0.241–5.530)	0.856	0.385 (0.020–7.404)	0.527	0.444 (0.022–9.032)	0.598

#### 3.2.2 Second episode of *Clostridioides difficile* infection (CDI)

Out of the 156 patients who received treatment for their first episode, 56 experienced a second episode. Of these, only 49 (87.50 %) were treated. The data are shown in the Supplementary Fig. 2. In addition, non-statistically differences for cure either re-infection were found comparing treatments after the second episode of CDI.

#### 3.2.3 Multiple rCDI

A third episode of CDI was experienced by 12 of the 49 patients treated during the second episode. Out of those, only eight received treatment, with three receiving vancomycin and five receiving fidaxomicin. When comparing the effectiveness of these treatments for this episode, no statistically significant differences were observed [OR: 0.500 (95%IC: 0.019–12.898); *p* = 0.676].

Only two patients suffered a fourth episode, which was treated with vancomycin.

### 3.3 Evolution of antimicrobial resistance of *Clostridioides difficile*

A total of seven samples (4.07 %) were resistant to vancomycin, whereas six samples (3.49 %) were resistant to metronidazole, including five samples (2.91 %) which were resistant to both.

We compared the MIC of vancomycin and metronidazole for September of 2022 and June of 2023, both months with more than ten strains evaluated and not resistances detected. Vancomycin resistance decreased statistically (*p* = 0.003, Mann-Whitney test), while the resistance against metronidazole did not change (*p* = 0.103) (Table 5 and Supplementary Fig. 3).

**Table 5:** Median values of Minimum Inhibitory Concentrations (MICs) of *Clostridiodes difficile* to vancomycin and metronidazole.

**Median**	**June 2022**	**July 2022**	**August 2022**	**September 2022**	**October 2022**	**November 2022**	**December 2022**
**MIC Metronidazole**	0.125	0.050	0.055	0.060	0.090	0.125	0.158
	(0.125–0.125)	(0.030–16.030)	(0.050–0.068)	(0.057–0.091)	(0.090–0.250)	(0.090–0.380)	(0.090–0.283)
**Nº MTZ resistance strain**	0	1	2	0	0	0	0
**Nºsamples**	2	5	14	10	13	10	10
**MIC Vancomycin**	0.380 (0.380–0.380)	0.250 (0.0220–128.125)	0.250 (0.190–0.410)	0.220 (0.084–0.250)	0.125 (0.092–0.250)	0.253 (0.125–0.380)	0.188 (0.117–0.283)
**Nº VAN resistance strain**	0	1	2	0	0	0	0

**January 2023**	**February 2023**	**March 2023**	**April 2023**	**May 2023**	**June 2023**
0.125(0.091–0.380)	0.190 (0.125–0.250)	0.130 (0.090–0.250)	0.125 (0.090–0.380)	0.125 (0.099–0.380)	0.125 (0.060–0.190)
2	0	1	0	0	0
20	15	27	15	20	11
0.380 (0.250–0.500)	0.190 (0.125–0.250)	0.250 (0.125–0.380)	0.250 (0.094–0.380)	0.380 (0.141–0.470)	0.380 (0.250–0.500)
2	0	2	0	0	0

### 3.4 Hypervirulent CDI

Ribotype 027 was identified in 17 (8.76 %) patients with one recurrence and a mortality rate of 29.41 % (5/17). Hypervirulent *C. difficile* was not associated with either death (*p* = 0.202) or rCDI (*p* = 0.221).

## 4 Discussion

Demographic characteristics show variations between the CDI treatment groups. We observed a different pattern among patients receiving different treatments. Initially, metronidazole was the primary treatment for CDI; however, it is no longer recommended [7]. Currently, fidaxomicin or vancomycin is preferred, with vancomycin being the alternative when fidaxomicin is not available [7].

Patients treated with metronidazole were younger, less hospitalised and had fewer comorbidities and non-gastrointestinal infection. However, they experienced a higher proportion of gastrointestinal coinfections. In contrast, vancomycin-treated patients were older, suffered more non-gastrointestinal coinfections and had a higher rate of hospitalization with longer hospital stays. They, also experienced fewer gastrointestinal coinfections. Hospital policy tends to use fidaxomicin for patients with recurrences or a high risk of death or such as hospitalised elderly with many comorbidities. This group of patients is usually composed of nosocomial CDI. Patients with combination therapy showed a similar trend to the fidaxomicin group.

These discrepancies may be attributed to the severity of the infection, which was not included in our study. Probably, younger individuals received metronidazole and were not hospitalised because they did not have any additional symptoms. This reasoning can be extended to the other treatments, as they are generally prescribed to patients in more critical health conditions.

In this single-centre retrospective cohort study, we found that fidaxomicin as initial treatment for CDI was a risk factor for recurrence, whereas patients treated with vancomycin and metronidazole showed no difference with the variable studied [9]. In fact, our results are in agreement with the investigation by Alhameed et al., 2023 [23] in which patients treated with vancomycin or metronidazole had similar treatment responses (96.4 % versus 94.3 %, *p* = 0.682) for non-fulminant CDI. However, those receiving vancomycin had a better early response with statistically significant differences (94.0 % versus 77.8 %, *p* = 0.008), which may have been due to the fact that vancomycin kills these bacteria faster than metronidazole because of its higher faecal concentrations [23]. However, others studies found in the literature differ from these, where fidaxomicin treatment was better for first incidence of CDI, first recurrence and non-severe CDI than vancomycin and metronidazole [11,24]. In the investigation by Polivkova et al.*,* 2021 [11], fidaxomicin had higher sustained cure and lower recurrence than vancomycin and metronidazole. Other studies [[24], [25], [26]] also found better sustained cure and lower recurrence rates for fidaxomicin compared to vancomycin. This is because, unlike metronidazole or vancomycin, fidaxomicin inhibits *C. difficile* sporulation [27]. However, in the meta-analysis by Dai et al., 2022 [9], in which they studied ten different studies comparing these same three treatment, they found no significant improvement with any of them, and that there was a significantly higher risk of therapeutic failure in patients with CDI treated with fidaxomicin [9].

Dai et al. (2022) highlighted that many of the trials used for meta-analysis were conducted before the dissemination of the 2017 IDSA/SHEA guidelines, which supported the abbreviated 10-day vancomycin regimen [7,9]. However, assessment of treatment efficacy in clinical trials is usually performed in strictly controlled settings, which may lead to conclusions that differ from real-world scenarios. Therefore, analyses such as the one presented by Dai et al.*,*. (2022) are essential for understanding treatment outcomes in real-world patient populations [9]. A major factor contributing to the limited use of fidaxomicin for treatment [7,9,11] or for CDI research is its cost [8]. Therefore, further research involving larger cohorts of patients treated with fidaxomicin is needed to strengthen the understanding of fidaxomicin in the first episode of CDI.The second episode of CDI was analysed without statistical difference, while in other studies, fidaxomicin remained a better treatment for this recurrence [11]. The reason for this could be that this drug has limited activity against the gut microbiota. Therefore, this results in CDI patients having a better sustained clinical response and a lower recurrence rate [9].

Twelve patients had a third episode of CDI. Only eight of them were treated, three with vancomycin and five with fidaxomicin. Two of the patients suffered a fourth episode that was treated with vancomycin despite guidelines stipulating that after a recurrence of CDI, fidaxomicin should be the prescribed treatment [7].

Regarding antimicrobial resistance, 4.07 % of samples were resistant to vancomycin and 3.49 % to metronidazole, most of them being sensitive to both. Our results are in agreement with several studies conducted in Europe and the United States [17,28]. The study by Abdrabou et al. (2019) [28] found 0 % vancomycin and 2.7 % metronidazole resistance in isolates from the German National Reference Centre between 2014 and 2019, while 0 % and 0.2 % resistances were found in isolates from a German tertiary hospital in the same period. Furthermore, the study by Boyánova et al. (2023) [17] analysed antimicrobial resistance of *C. difficile* in 20 countries. Resistance rates to metronidazole and vancomycin were found to be 4.7 % and 2.6 %, respectively, being higher rates for other antibiotics such as rifampicin, erythromycin, and imipenem. The surveillance study by Snydman et al.*,* (2023) [29] conducted in the United States between 2020 and 2021 observed resistance to vancomycin in 0.7 % of isolates according to CLSI and EUCAST criteria and to metronidazole in 0.3 % according to EUCAST criteria. However, despite the low resistance to these two antibiotics shown by *C. difficile,* continued surveillance is necessary due to increasing antimicrobial resistance and the introduction of hypervirulent ribotypes such as 027.

MICs for vancomycin resistance showed a decrease. One of the reasons may be an increase in the use of fidaxomicin, the recommended antibiotic treatment for CDIs [7].

Antimicrobial resistance is a serious public health problem, recognized by the World Health Organization (WHO), which is associated with severe socio-economic consequences [30]. For this reason, new therapeutic strategies, such as new small molecules, natural products, antitoxin monoclonal antibodies, bacteriophages or fecal microbiota transplantation, are urgently needed [31]. One of the most promising small molecules tested is ridinilazole, which is a likely narrow-spectrum antibiotic associated with minor changes in gut microbiota in patients with CDI [32]. These findings are supported by several clinical trials such as Vickers et al. (2017) [33], which demonstrated non-inferiority of ridinilazole versus vancomycin by presenting statistically significant sustained clinical efficacy [Treatment difference: 21.1 %, 90 % CI: 3.1–39.1 %, *p* = 0.0004). In conclusion, further studies are needed to increase the therapeutic arsenal against this bacterium.

Hypervirulent *C. difficile* strains predispose to a worse clinical outcome because they are associated with higher virulence factors, such as greater sporulation and toxin production and synthesis of a more lethal toxin B than other ribotypes [7,34,35]. However, different studies show that these strains, as in the case of ribotype 027 was associated with a better prognosis when compared to ribotype 106 [36]. Some authors, such as Martin et al.*,* (2018) [37], described that CDI of ribotype 027 tended to be health area clones with the same clinical effects as normal strains [37].

Limitations of this study include the fact that it was designed as a single-centre study and the number of patients in some treatment groups is reduced. In addition, study did not assess severity of infection, which could have helped with the differences in the demographic variables for each treatment. Another limitation is that some patients who had their first episode in 2014 did not receive the treatment recommended by the current guidelines because fidaxomicin was not available, which reduced the number of samples for the antimicrobial resistance study [38,39]. In addition, the MIC gradient strip test gives a result that may differ depending on the investigator that collecting the data and only metronidazole and vancomycin susceptibility was tested because a commercial fidaxomicin was not test available. In fact, we found that patients treated with fidaxomicin were older and had more comorbidities. These factors could have influenced the results obtained in our study, as fidaxomicin is used in complex chronic patients. Finally, the few cases of hypervirulent *C. difficile* strains may have biased the statistical analysis with an inconclusive result on the possible implication of this ribotype in CDI.”

## 5 Conclusion

In the cohort of patients studied, metronidazole were superior to fidaxomicin for the second episode of rCDI. This may be influenced by the fact that fidaxomicin is reserved for patients with higher risk factors. To resolve this, future prospective randomised studies are needed to confirm these findings and thus optimise treatment in groups of patients with multiple rCDI.

Antibiotic resistance should be monitored periodically to detect early the emergence of strains resistant to recommended treatments. More hypervirulent strains of *C. difficile* needs to be studied to recognize possible related outcomes.

## 6 Compliance with ethics guidelines

The study was conducted in accordance with the protocol and the principles set out in the current revised version of the Declaration of Helsinki (Fortaleza, October 2013) and in accordance with applicable regulatory requirements, in particular the ICH Tripartite Harmonised Standards for Good Clinical Practice 1996 and approved by the Ethics Committee of Hospital Universitario Príncipe de Asturias (Madrid) (Ref. OE May 2023; March 29, 2023).

Due to the retrospective nature of the study, the Ethics Committee determined that no patient consent was required.

## Data availability statement

Data cannot be shared publicly because they are confidential. Data are available from the Clinical Microbiology Department of the Hospital Universitario Príncipe de Asturias for researchers who meet the criteria to access confidential data.

## Funding

This work form part of the research project “The search for new compounds with antimicrobial properties against multidrug-resistant pathogens or within effective treatments” from Hospital Universitario Príncipe de Asturias.

## CRediT authorship contribution statement

**Marcos Hernando-Gozalo:** Writing – review & editing, Writing – original draft, Visualization, Validation, Methodology, Investigation, Formal analysis, Data curation, Conceptualization. **Carlos Rescalvo-Casas:** Writing – review & editing, Writing – original draft, Visualization, Validation, Supervision, Methodology, Investigation, Formal analysis, Data curation, Conceptualization. **Laura Seijas-Pereda:** Writing – review & editing, Writing – original draft, Investigation. **Juan Cuadros-González:** Writing – review & editing, Writing – original draft, Visualization, Validation, Supervision, Conceptualization. **Ramón Pérez-Tanoira:** Writing – review & editing, Writing – original draft, Visualization, Validation, Supervision, Investigation, Formal analysis, Data curation, Conceptualization.

## Declaration of competing interest

The authors declare that they have no known competing financial interests or personal relationships that could have appeared to influence the work reported in this paper.

## References

[1] Czepiel J., Dróżdż M., Pituch H., Kuijper E.J., Perucki W., Mielimonka A., Goldman S., Wultańska D., Garlicki A., Biesiada G. (2019). Clostridium difficile infection: review. Eur. J. Clin. Microbiol. Infect. Dis..

[2] Brown N.A., Lebar W.D., Young C.L., Hankerd R.E., Newton D.W. (2011). Diagnosis of Clostridium difficile infection: comparison of four methods on specimens collected in Cary-Blair transport medium and tcdB PCR on fresh versus frozen samples. Infect. Dis. Rep..

[3] Alcalá-Hernández L., Mena-Ribas A., Niubó-Bosh J., Marín-Arriaza M. (2016). Diagnóstico microbiológico de la infección por Clostridium difficile. Enferm. Infecc. Microbiol. Clín..

[4] ECDC, European Centre for Disease Prevention and Control. Healthcare-associated Infections: Clostridium difficile Infections, Stockholm, 2018.

[5] J. Mensa Pueyo, A. Soriano Viladomiu, E. Lopez Sune, Y. Zboromyrska, P. Llinanres Mondejar, P. Barberan Lopez, Guia de terapeutica antimicrobiana 2023, Antares, Barcelona, 2023.

[6] van Prehn J., Reigadas E., Vogelzang E.H., Bouza E., Hristea A., Guery B., Krutova M., Norén T., Allerberger F., Coia J.E., Goorhuis A., van Rossen T.M., Ooijevaar R.E., Burns K., Scharvik Olesen B.R., Tschudin-Sutter S., Wilcox M.H., Vehreschild M.J.G.T., Fitzpatrick F., Kuijper E.J. (2021). Guideline Committee of the European study group on Clostridioides difficile, European Society of clinical Microbiology and infectious diseases: 2021 update on the treatment guidance document for Clostridioides difficile infection in adults. Clin. Microbiol. Infect..

[7] Johnson S., Lavergne V., Skinner A.M., Gonzales-Luna A.J., Garey K.W., Kelly C.P., Wilcox M.H. (2021). Clinical practice guideline by the infectious diseases Society of America (IDSA) and Society for Healthcare epidemiology of America (SHEA): 2021 Focused update guidelines on Management of *Clostridioides difficile* infection in adults. Clin. Infect. Dis..

[8] Najjar-Debbiny R., Bazazhina A., Schwartz N., Shaked P., Saliba W., Weber G. (2022). Non-inferiority of metronidazole to vancomycin in the treatment of first episode non-severe Clostridioides difficile infection: a single center retrospective cohort study. Infection.

[9] Dai J., Gong J., Guo R. (2022). Real-world comparison of fidaxomicin versus vancomycin or metronidazole in the treatment of Clostridium difficile infection: a systematic review and meta-analysis. Eur. J. Clin. Pharmacol..

[10] Igarashi Y., Tashiro S., Enoki Y., Taguchi K., Matsumoto K., Ohge H., Suzuki H., Nakamura A., Mori N., Morinaga Y., Yamagishi Y., Yoshizawa S., Yanagihara K., Mikamo H., Kunishima H. (2018). Oral vancomycin versus metronidazole for the treatment of Clostridioides difficile infection: meta-analysis of randomized controlled trials. J. Infect. Chemother..

[11] Polivkova S., Krutova M., Capek V., Sykorova B., Benes J. (2021). Fidaxomicin versus metronidazole, vancomycin and their combination for initial episode, first recurrence and severe Clostridioides difficile infection — an observational cohort study. Int. J. Infect. Dis..

[12] Bishop E.J., Tiruvoipati R. (2023). Management of *Clostridioides difficile* infection in adults and challenges in clinical practice: review and comparison of current IDSA/SHEA, ESCMID and ASID guidelines. J. Antimicrob. Chemother..

[13] Okumura H., Fukushima A., Taieb V., Shoji S., English M. (2020). Fidaxomicin compared with vancomycin and metronidazole for the treatment of Clostridioides (Clostridium) difficile infection: a network meta-analysis. J. Infect. Chemother..

[14] Shen W.-J., Deshpande A., Hevener K.E., Endres B.T., Garey K.W., Palmer K.L., Hurdle J.G. (2020). Constitutive expression of the cryptic vanGCd operon promotes vancomycin resistance in Clostridioides difficile clinical isolates. J. Antimicrob. Chemother..

[15] Marchandin H., Anjou C., Poulen G., Freeman J., Wilcox M., Jean-Pierre H., Barbut F. (2023). *In vivo* emergence of a still uncommon resistance to fidaxomicin in the urgent antimicrobial resistance threat *Clostridioides difficile*. J. Antimicrob. Chemother..

[16] Sholeh M., Krutova M., Forouzesh M., Mironov S., Sadeghifard N., Molaeipour L., Maleki A., Kouhsari E. (2020). Antimicrobial resistance in Clostridioides (Clostridium) difficile derived from humans: a systematic review and meta-analysis. Antimicrob. Resist. Infect. Control.

[17] Boyanova L., Dimitrov G., Gergova R., Hadzhiyski P., Markovska R. (2023). *Clostridioides difficile* resistance to antibiotics, including post-COVID-19 data. Expert Rev Clin Pharmacol.

[18] Madden G.R., Poulter M.D., Sifri C.D. (2019). PCR cycle threshold to assess a diagnostic stewardship intervention for C. difficile testing. J. Infect..

[19] Red Nacional de Vigilancia Epidemiológica (RENAVE) (2016).

[20] Merchante N., Herrero R., Valverde-Fredet M.D., Rodríguez-Fernández M., Pinargote H., Martínez-Marcos F.J., Gil-Anguita C., García-López M., Tasias Pitarch M., Abril López De Medrano V., Navarrete Lorite M.N., Gómez-Ayerbe C., León E., González-De La Aleja P., Ruiz Castillo A., Aller A.I., Rodríguez J.C., Ternero Fonseca J., Corzo J.E., Naranjo Pérez A., Trigo-Rodríguez M., Merino E. (2023). Role of previous systemic antibiotic therapy on the probability of recurrence after an initial episode of *Clostridioides difficile* infection treated with vancomycin. JAC Antimicrob Resist.

[21] Schwartz O., Rohana H., Azrad M., Shor A., Rainy N., Maor Y., Nesher L., Sagi O., Ken-Dror S., Kechker P., Peretz A. (2023). Characterization of community-acquired Clostridioides difficile strains in Israel, 2020-2022. Front. Microbiol..

[22] The European Committee on Antimicrobial Susceptibility Testing (2023). Breakpoint tables for interpretation of MICs and zone diameters. Versino 13.1, 2023. http://www.eucast.org.

[23] Alhameed A.F., Saferuddin N., Alturkistani T., Al Musawa M., Damfu N., Alattas M. (2023). Vancomycin vs metronidazole use for the treatment of Clostridioides difficile infection in a tertiary care hospital in Saudi Arabia. Heliyon.

[24] Skally M., Bennett K., Burns K., Brennan R., Finn C., O'Connell K., Dinesh B., O'Donnell S., Fawley W., Wilcox M., Humphreys H., Fitzpatrick F. (2023). A decade of Clostridioides difficile infection: a constant challenge to maintain the status quo. J. Hosp. Infect..

[25] Long B., Gottlieb M. (2022). Oral fidaxomicin versus vancomycin for *Clostridioides difficile* infection. Acad. Emerg. Med..

[26] Tashiro S., Mihara T., Sasaki M., Shimamura C., Shimamura R., Suzuki S., Yoshikawa M., Hasegawa T., Enoki Y., Taguchi K., Matsumoto K., Ohge H., Suzuki H., Nakamura A., Mori N., Morinaga Y., Yamagishi Y., Yoshizawa S., Yanagihara K., Mikamo H., Kunishima H. (2022). Oral fidaxomicin versus vancomycin for the treatment of Clostridioides difficile infection: a systematic review and meta-analysis of randomized controlled trials. J. Infect. Chemother..

[27] Babakhani F., Bouillaut L., Gomez A., Sears P., Nguyen L., Sonenshein A.L. (2012). Fidaxomicin inhibits Spore production in Clostridium difficile. Clin. Infect. Dis..

[28] Abdrabou A.M.M., Ul Habib Bajwa Z., Halfmann A., Mellmann A., Nimmesgern A., Margardt L., Bischoff M., von Müller L., Gärtner B., Berger F.K. (2021). Molecular epidemiology and antimicrobial resistance of Clostridioides difficile in Germany, 2014–2019. International Journal of Medical Microbiology.

[29] Snydman D.R., McDermott L.A., Thorpe C.M., Goldstein E.J.C., Schuetz A.N., Johnson S., Gerding D.N., Gluck L., Bourdas D., Carroll K.C., Lancaster C.K., Garey K.W., Wang Q., Walk S.T., Duperchy E. (2023). A US-based national surveillance study for the susceptibility and epidemiology of *Clostridioides difficile* isolates with special reference to ridinilazole: 2020–2021. Antimicrob. Agents Chemother..

[30] Gil‐Gil T., Laborda P., Sanz‐García F., Hernando‐Amado S., Blanco P., Martínez J.L. (2019). Antimicrobial resistance: a multifaceted problem with multipronged solutions. Microbiologyopen.

[31] Phanchana M., Harnvoravongchai P., Wongkuna S., Phetruen T., Phothichaisri W., Panturat S., Pipatthana M., Charoensutthivarakul S., Chankhamhaengdecha S., Janvilisri T. (2021). Frontiers in antibiotic alternatives for *Clostridioides difficile* infection. World J. Gastroenterol..

[32] Thorpe C.M., Kane A.V., Chang J., Tai A., Vickers R.J., Snydman D.R. (2018). Enhanced preservation of the human intestinal microbiota by ridinilazole, a novel Clostridium difficile-targeting antibacterial, compared to vancomycin. PLoS One.

[33] Vickers R.J., Tillotson G.S., Nathan R., Hazan S., Pullman J., Lucasti C., Deck K., Yacyshyn B., Maliakkal B., Pesant Y., Tejura B., Roblin D., Gerding D.N., Wilcox M.H., Bhan A., Campbell W., Chopra T., Deck K., Golan Y., Gordon I., Kamepalli R., Khanna S., Lee C., Lucasti C., Maliakkal B., Minang I., Mullane K., Nathan R., Oughton M., Pesant Y., Phillips J., Pullman J., Riska P., Schrock C., Siegel J., Steinberg A., Talan D., Tamang S., Tan M., Weiss K., Wang C., Yacyshyn B., Young J.-A., Zenilman J. (2017). Efficacy and safety of ridinilazole compared with vancomycin for the treatment of Clostridium difficile infection: a phase 2, randomised, double-blind, active-controlled, non-inferiority study. Lancet Infect. Dis..

[34] Martínez-Meléndez A., Morfin-Otero R., Villarreal-Treviño L., Baines S.D., Camacho-Ortíz A., Garza-González E. (2020). Molecular epidemiology of predominant and emerging Clostridioides difficile ribotypes. J. Microbiol. Methods.

[35] Rao K., Micic D., Natarajan M., Winters S., Kiel M.J., Walk S.T., Santhosh K., Mogle J.A., Galecki A.T., LeBar W., Higgins P.D.R., Young V.B., Aronoff D.M. (2015). *Clostridium difficile* ribotype 027: Relationship to age, Detectability of toxins A or B in stool with rapid testing, severe infection, and mortality. Clin. Infect. Dis..

[36] Almutairi M.S., Gonzales-Luna A.J., Alnezary F.S., Fallatah S.B., Alam M.J., Begum K., Garey K.W. (2021). Comparative clinical outcomes evaluation of hospitalized patients infected with Clostridioides difficile ribotype 106 vs. other toxigenic strains. Anaerobe.

[37] Martin J.S.H., Eyre D.W., Fawley W.N., Griffiths D., Davies K., Mawer D.P.C., Peto T.E.A., Crook D.W., Walker A.S., Wilcox M.H. (2018). Patient and strain characteristics associated with Clostridium difficile Transmission and Adverse outcomes. Clin. Infect. Dis..

[38] McDonald L.C., Gerding D.N., Johnson S., Bakken J.S., Carroll K.C., Coffin S.E., Dubberke E.R., Garey K.W., V Gould C., Kelly C., Loo V., Shaklee Sammons J., Sandora T.J., Wilcox M.H. (2018). Clinical practice guidelines for Clostridium difficile infection in adults and Children: 2017 update by the infectious diseases Society of America (IDSA) and Society for Healthcare epidemiology of America (SHEA). Clin. Infect. Dis..

[39] Cohen S.H., Gerding D.N., Johnson S., Kelly C.P., Loo V.G., McDonald L.C., Pepin J., Wilcox M.H. (2010). Clinical practice guidelines for Clostridium difficile infection in adults: 2010 update by the Society for Healthcare epidemiology of America (SHEA) and the infectious diseases Society of America (IDSA). Infect. Control Hosp. Epidemiol..

