# Supplementary material for: Comparison of fidaxomicin, metronidazole and vancomycin for initial episode and recurrence of Clostridioides difficile infection - An observational cohort study
Source: Heliyon. 2024 May 7;10(10):e30742. doi: 10.1016/j.heliyon.2024.e30742 (PMC11128465; doi:10.1016/j.heliyon.2024.e30742)
Supplement: Multimedia component 1 [file mmc1.docx]

**B**

**A**

**%**

|  | **MTZ** | **VAN** | **FDX** | **MTZ+ VAN** |  | **MTZ** | **VAN** | **FDX** | **MTZ+ VAN** |
| --- | --- | --- | --- | --- | --- | --- | --- | --- | --- |
| **All patients** | **42** | **85** | **13** | **16** | **All patients** | **42** | **85** | **13** | **16** |
| **Sustained cure** | **21** | **42** | **3** | **6** | **rCDI** | **11** | **31** | **8** | **6** |
|  |  |  |  |  | **Died** | **10** | **12** | **2** | **4** |

*****

*****

**49.41**

**61.54**

**37.50**

**25.00**

**26.19**

**36.47**

**37.50**

**50.00**

**Supplementary Figure 1.** First episode of *Clostridioides difficile* infection (CDI): (A) Sustained cure against CDI, (B) second episode of CDI. **Blue for MTZ**: metronidazole; **Red for VAN**: vancomycin; **Yellow for FDX**: fidaxomicin; **Green for MTZ+VAN**: Combination. *Statisticial difference.
