# Supplementary material for: Comparison of fidaxomicin, metronidazole and vancomycin for initial episode and recurrence of Clostridioides difficile infection - An observational cohort study
Source: Heliyon. 2024 May 7;10(10):e30742. doi: 10.1016/j.heliyon.2024.e30742 (PMC11128465; doi:10.1016/j.heliyon.2024.e30742)
Supplement: Multimedia component 2 [file mmc2.docx]

|  | **MTZ** | **VAN** | **FDX** | **MTZ+ VAN** |  | **MTZ** | **VAN** | **FDX** | **MTZ+ VAN** |
| --- | --- | --- | --- | --- | --- | --- | --- | --- | --- |
| **All patients** | **7** | **25** | **15** | **2** | **All patients** | **7** | **25** | **15** | **2** |
| **Sustained cure** | **3** | **14** | **6** | **1** | **rCDI** | **1** | **5** | **5** | **1** |
|  |  |  |  |  | **Died** | **3** | **6** | **4** | **0** |

**50.00**

**50.00**

**56.00**

**40.00**

**26.67**

**20.00**

**14.29**

**42.86**

**A**

**B**

**%**

**Supplementary Figure 2.** Second episode of *Clostridioides difficile* infection (CDI): (A) Sustained cure against second episode of CDI, (B) third episode of CDI. **Blue for MTZ**: metronidazole; **Red for VAN**: vancomycin; **Yellow for FDX**: fidaxomicin; **Green for MTZ+VAN**: Combination.
