# Supplementary material for: Comparison of fidaxomicin, metronidazole and vancomycin for initial episode and recurrence of Clostridioides difficile infection - An observational cohort study
Source: Heliyon. 2024 May 7;10(10):e30742. doi: 10.1016/j.heliyon.2024.e30742 (PMC11128465; doi:10.1016/j.heliyon.2024.e30742)
Supplement: Multimedia component 3 [file mmc3.docx]

0.30

0.20

0.10

0.15

0.05

0.25

0.35

0.4

**Supplementary Figure 3.** Changes in the antimicrobial susceptibility profile of *Clostridioides difficile* isolates observed between June 2022 and June 2023**.** Mann-Whitney test was performed to obtain statistical median differences**.**
